# Production of highly pure *R,R*-2,3-butanediol for biological plant growth promoting agent using carbon feeding control of *Paenibacillus polymyxa* MDBDO

**DOI:** 10.1186/s12934-023-02133-y

**Published:** 2023-07-05

**Authors:** Jung-Hyun Ju, Min-Ho Jo, Sun-Yeon Heo, Min-Soo Kim, Chul-Ho Kim, Narayan Chandra Paul, Hyunkyu Sang, Baek-Rock Oh

**Affiliations:** 1 Microbial Biotechnology Research Center, Korea Research Institute of Bioscience and Biotechnology (KRIBB), Jeongeup, Jeonbuk 56212 Republic of Korea; 2 Department of Integrative Food, Bioscience and Biotechnology, Chonnam National University, Gwangju, 61186 Republic of Korea; 3 Kumho Life Science Laboratory, Chonnam National University, Gwangju, 61186 Republic of Korea

**Keywords:** Biological plant growth promoting agent, *Paenibacillus polymyxa*, *R,R*-2,3-Butanediol, Optical purity, Carbon feeding control

## Abstract

**Background:**

Chemical fertilizers have greatly contributed to the development of agriculture, but alternative fertilizers are needed for the sustainable development of agriculture. 2,3-butanediol (2,3-BDO) is a promising biological plant growth promoter.

**Results:**

In this study, we attempted to develop an effective strategy for the biological production of highly pure *R,R*-2,3-butanediol (*R,R*-2,3-BDO) by *Paenibacillus polymyxa* fermentation. First, gamma-ray mutagenesis was performed to obtain *P. polymyxa* MDBDO, a strain that grew faster than the parent strain and had high production of *R,R*-2,3-BDO. The activities of *R,R-*2,3-butanediol dehydrogenase and diacetyl reductase of the mutant strain were increased by 33% and decreased by 60%, respectively. In addition, it was confirmed that the carbon source depletion of the fermentation broth affects the purity of *R,R*-2,3-BDO through batch fermentation. Fed-batch fermentation using controlled carbon feeding led to production of 77.3 g/L of *R,R*-2,3-BDO with high optical purity (> 99% of C_4_ products) at 48 h. Additionally, fed-batch culture using corn steep liquor as an alternative nitrogen source led to production of 70.3 g/L of *R,R*-2,3-BDO at 60 h. The fed-batch fermentation broth of *P. polymyxa* MDBDO, which contained highly pure *R,R*-2,3-BDO, significantly stimulated the growth of soybean and strawberry seedlings.

**Conclusions:**

This study suggests that *P. polymyxa* MDBDO has potential for use in biological plant growth promoting agent applications. In addition, our fermentation strategy demonstrated that high-purity *R,R*-2,3-BDO can be produced at high concentrations using *P. polymyxa*.

**Supplementary Information:**

The online version contains supplementary material available at 10.1186/s12934-023-02133-y.

## Background

An increase in global food demand due to a growing population has led to the indiscriminate use of chemical fertilizers and other agrochemicals to increase agricultural yields [1]. Chemical fertilizer was a groundbreaking invention that dramatically increased agricultural production, but caused groundwater and seawater pollution and soil acidification due to eutrophication of the soil [2]. In addition, the increase in nitrous oxide caused by the use of chemical fertilizers affects global warming [3]. In order to solve these problems of chemical fertilizers, various researches on alternative plant growth promoting agents are in progress [4–7]. Among them, a promising alternative plant growth promoting agent is 2,3-butanediol (2,3-BDO). 2,3-BDO is an odorless, colorless, liquid organic compound with the formula (CH_3_CHOH)_2_ [8]. Several previous studies have reported that 2,3-BDO has a plant growth promoting effect [5, 6, 9, 10]. 2,3-BDO exists in three isomers: the *levo* form (*2R,3R*), the *dextro* form (*2S,3S*), and the optically inactive *meso* form (*2R,3S*). Most of the studies on the plant growth promoting effect of 2,3-BDO have not been done in terms of isomers. Nonetheless, among the three isomers of 2,3-BDO, *R,R*-2,3-BDO is well known for its plant growth promoting effect [5, 6] and anti-fungal effect [11, 12]. Depending on the type of plant, *R,R*-2,3-BDO and *meso*-2,3-BDO have been reported to have plant virus resistance effects [13]. 2,3-BDO and its derivatives can be utilized as fuels, solvents, cosmetics, foods, drugs, plastics, and pesticides, depending on chirality [14, 15]. 2,3-BDO can be produced by the hydrolysis of 2,3-epoxybutane, and the percentage of different isomer products is determined by the stereochemistry of the epoxide [16]. Commercially, chemical production of 2,3-BDO requires high temperature and pressure (160–220 °C, 50 bar), and can lead to the production of many by-products, depletion of petroleum resources, and environmental pollution [17].

An alternative approach is the production of 2,3-BDO using microorganisms. Previous studies reported production of microbial 2,3-BDO in various microbial species, including *Klebsiella* sp. [18, 19], *Enterobacter* sp. [20, 21], and *Serratia* sp. [22], all of which are biosafety level 2 (BSL-2) microorganisms (moderate hazards). The most promising strain available for industrial use is *K. pneumoniae* because it can produce the highest 2,3-BDO concentration, about 150 g/L [23]. However, the pathogenicity of *Klebsiella* and its ability to cause opportunistic infections are generally considered obstacles to its use for the large-scale production of 2,3-BD [24]. Certain non-pathogenic (BSL-1) microorganisms, such as *Bacillus subtilis* [25], *B. licheniformis* [26], *B. amyloliquefaciens*, and *Paenibacillus* can also produce 2,3-BDO with high efficiency. *P. polymyxa* in particular has potential for the industrial production of *R,R*-2,3-BD [27, 28]. Previous research reported the highest 2,3-BDO concentration obtained by *P. polymyxa* DSM 365 was 111 g/L when using a high concentration organic nitrogen source [29]. Although, this method produced a high concentration of *R,R*-2,3-BDO was produced, the optical purity was 98% (w/w).

2,3-BDO has a variety of commercial uses. In particular, it is used as an agricultural control agent because it has antimicrobial properties against the bacterial, fungal, and viral pathogens [5, 13, 30] and it also promotes plant growth [5, 6]. In addition, highly pure *R,R*-2,3-BDO is essential for certain applications, such as high value-added pharmaceuticals and liquid crystals [31, 32]. During microbial diol production, the separation of diols from the fermentation broth accounts for more than 50% of the total cost [33]. Therefore, it is necessary to achieve high concentration and purity for the efficient industrial production of *R,R*-2,3-BDO. A recent study reported that a genetically modified strain of *P. polymyxa* produced *R,R*-2,3-BDO that had high optical purity [34]. However, commercial use of this strain may be problematic because it has antibiotic resistance and is a genetically modified organism (GMO) [35].

Previous studies that examined the chiral synthesis of *R,R*-2,3-BDO by *P. polymyxa* examined the effect of adding different chemicals (e.g., acetic acid) or changing fermentation conditions (e.g., dilution rate, pH, oxygen supply) [29, 36–38]. Many of these studies demonstrated the importance of controlling oxygen availability to reduce the production of *meso*-2,3-BDO. Thus, there has been a focus on factors that impact the oxygen transfer coefficient, such as agitation speed and aeration rate. For example, Hassler et al. controlled the agitation speed and aeration rate to prevent the formation of *meso*-2,3-BDO and increase the optical purity of *R,R*-2,3-BDO [29]. However, this strategy was not effective in controlling the production of acetoin.

*Paenibacillus polymyxa* converts *R*-acetoin into *R,R*-2,3-BDO using acetoin reductase/2,3-butanediol dehydrogenase (AR/2,3-BDH) with NADH as a coenzyme. Importantly, the AR/2,3-BDH from *P. polymyxa* is reversible. Thus, Gao et al. reported the *K*_*m*_ value of this enzyme from *P. polymyxa* ZJ-9 was about 38 times higher for *R*-acetoin than for *R,R*-2,3-BDO [39]. However, substrate affinity varies according to fermentation conditions and the external environment [40, 41]. It is important to maintain a high acetoin reduction activity of this reversible enzyme for the efficient production of *R,R*-2,3-BDO.

High-concentration production of high-purity *R,R*-2,3-BDO using *P. polymyxa* uses a high-concentration organic nitrogen source [29]. Nitrogen plays an important role in the growth of microorganisms during fermentation [42]. An appropriate carbon/nitrogen (C/N) ratio can enhance the growth of microorganisms and increase the productivity and titer of the desired product [43]. However, the use of high-concentration nitrogen sources can be an obstacle to industrialization of 2,3-BDO derived from microorganisms. Corn steep liquor (CSL), a by-product of the starch industry, is an inexpensive nitrogen source that can be used for microbial fermentation because it contains a rich complement of nitrogenous compounds, vitamins, amino acids, and biotins [44, 45]. The price of CSL is generally more than 30 times less than industrial yeast extract [43]. Therefore, CSL can be used as an excellent industrial alternative nitrogen source.

Our general purpose was to develop an effective strategy for the biological production of highly pure *R,R*-2,3-BDO by *P. polymyxa* fermentation. We first performed gamma-ray mutagenesis to obtain a mutant strain of *P. polymyxa* that had better growth and *R,R*-2,3-BDO production than the parent strain. We then confirmed the effect of the concentration of the carbon source on the production of acetoin and *R,R*-2,3-BDO through batch fermentation studies. We also performed fed-batch fermentation studies to determine the most suitable concentration of the carbon source for the production of *R,R*-2,3-BDO that had high optical purity. We then examined the effect of using CSL as a low-cost nitrogen source on the production of a high concentration of *R,R*-2,3-BDO. Finally, we examined the effect of the fermentation broth from *P. polymyxa* on the growth of soybean and strawberry seedlings to confirm its potential as the biological plant growth promoting agent.

## Results and discussion

### *Paenibacillus polymyxa *MDBDO for production of optically pure *R,R*-2,3-BDO

**Fig. 1:**
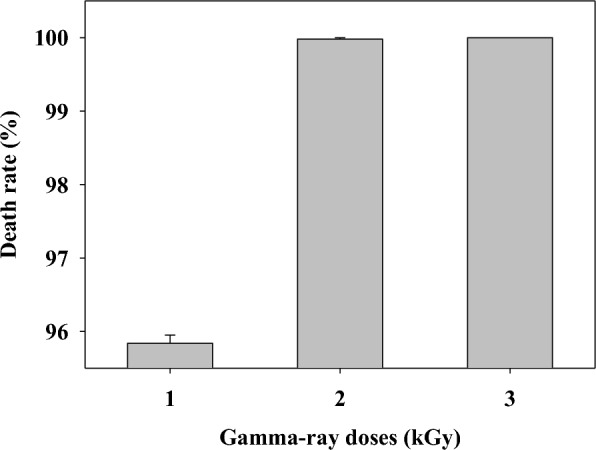
Effect of different gamma-ray doses on the death rate of *Paenibacillus polymyxa* DSM365 cells

Gamma-irradiation is a traditional method used for mutagenesis, and it remains useful in various industrial applications because the resulting strain is non-GMO and lacks antibiotic resistance. Therefore, we used gamma-irradiation to obtain a *P. polymyxa* mutant that had increased cell growth and production of *R,R*-2,3-BDO relative to the parent strain. The mortality rate of *P. polymyxa* DSM 365 increased with radiation dose, and was 100% when the dose was 3 kGy (Fig. 1). The mortality rate at a dose of 2 kGy was 99.98%, in that 1 out of about 5000 colonies survived. Spontaneous mutations in this species occur at a rate of 1/10^5^ to 1/10^8^ [46], and exposure to a mutagen can increase the mutation rate by more than 1000-fold. Thus, we used a radiation dose of 2 kGy to isolate a mutant strain that had an increased colony size.

We first grew the mutant and parent strains in flask using the 2,3-BDO production medium under the same conditions (Table 1). The mutant strain had higher rates of glucose consumption and cell growth. In addition, after 12 h, the parent strain produced 12.3 g/L of *R,R*-2,3-BDO and the mutant strain produced 15.4 g/L and neither strain produced detectable *meso*-2,3-BDO. We named this mutant strain *P. polymyxa* MDBDO.

**Table 1:** Flask culture cultivation parameters of the parent strain and mutant strain of *Paenibacillus polymyxa* at 12 h

Strain	Cell growth (OD_600 nm_)	Glucose consumption (g/L)	Acetoin (g/L)	*Meso*-2,3-BDO (g/L)	*R,R*-2,3-BDO (g/L)
*P. polymyxa* DSM365	14.2 ± 0.3	38.5 ± 1.1	0.7 ± 0.1	ND	12.3 ± 0.2
*P. polymyxa* MDBDO	16.8 ± 0.4	48.2 ± 1.3	0.8 ± 0.1	ND	15.4 ± 0.2

*ND* not detected

**Fig. 2:**
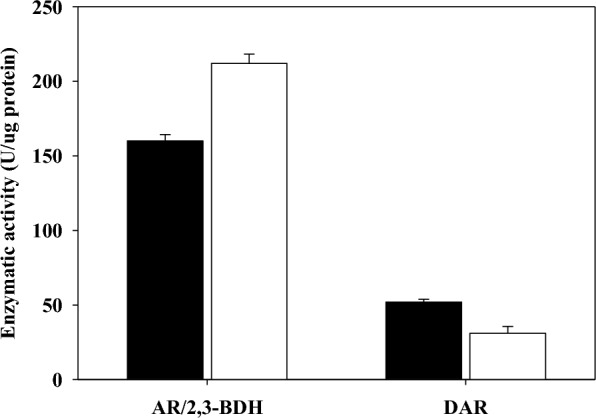
Acetoin reductase/2,3-butanediol dehydrogenase and diacetyl reductase enzymatic activity of the parent strain (black bars, *Paenibacillus polymyxa* DSM365) and the mutant strain (open bars, *Paenibacillus polymyxa* MDBDO)

To determine the cause of the increased *R,R*-2,3-BDO production by the mutant strain, we measured the activity of major enzymes involved in the production of *R,R*-2,3-BDO and *meso*-2,3-BDO (Fig. 2). The results showed that *P. polymyxa* MDBDO had 1.3-fold greater AR/2,3-BDH activity and the parent strain had 1.6-fold greater diacetyl reductase (DAR) activity. The increased AR/2,3-BDH activity and reduced DAR activity in the mutant were likely responsible for its increased production of *R,R*-2,3-BDO. Zhang et al. studied the same species and reported that *R,R*-2,3-BDO with an optical purity of 99.99% was obtained by removal of the DAR gene using genetic engineering [34]. DAR is the most important enzyme in the production of *meso*-2,3-BDO, because it produces *S*-2-acetoin (a precursor of *meso*-2,3-BDO) from the diacetyl that is produced by spontaneous decarboxylation. Although neither strain produced detectable *meso*-2,3-BDO, the decreased DAR activity in the mutant strain could contribute to the production of highly pure *R,R*-2,3-BDO. These changes in the activity of key enzymes that function in the production of 2,3-BDO demonstrate the potential value of *P. polymyxa* MDBDO for the industrial production of highly pure *R,R*-2,3-BDO.

### Batch fermentation of *P. polymyxa* MDBDO

**Fig. 3:**
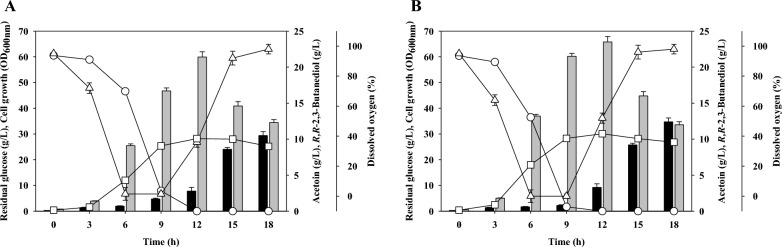
Batch fermentation of the parent strain (*Paenibacillus polymyxa* DSM365, **A**) and the mutant strain (*Paenibacillus polymyxa* MDBDO, **B**). Open circles, residual glucose; open squares, cell growth; open triangles, dissolved oxygen; black bars, acetoin; gray bars, *R,R*-2,3-butanediol

To examine the potential for microbial production of 2,3-BDO in an industrial setting, it is essential to perform batch fermentation experiments using fermentors. We therefore performed batch fermentation previous reported conditions (agitation speed: 500 rpm, aeration rate: 0.2 vvm) that led to the highest *R,R*-2,3-BDO production in a laboratory scale fermentor [29]. The batch fermentation results for the parent strain and *P. polymyxa* MDBDO show that both strains consumed all of the supplied carbon source by 12 h, and the cell growth at this time was OD_600 nm_ 28.2 in the parent strain and OD_600 nm_ 30.1 in the mutant (Fig. 3). The parent strain produced the most *R,R*-2,3-BDO (21.4 g/L) at 12 h and produced no detectable *meso*-2,3-BDO. The mutant strain produced the most *R,R*-2,3-BDO (23.5 g/L) at 12 h, and also produced no detectable *meso*-2,3-BDO. In addition, the maximum productivity of *R,R*-2,3-BDO was 1.86 g/L/h in the parent strain and 2.39 g/L/h in the mutant strain at 9 h. Thus, *R,R*-2,3-BDO productivity was about 1.3-times higher in *P. polymyxa* MDBDO.

**Fig. 4:**
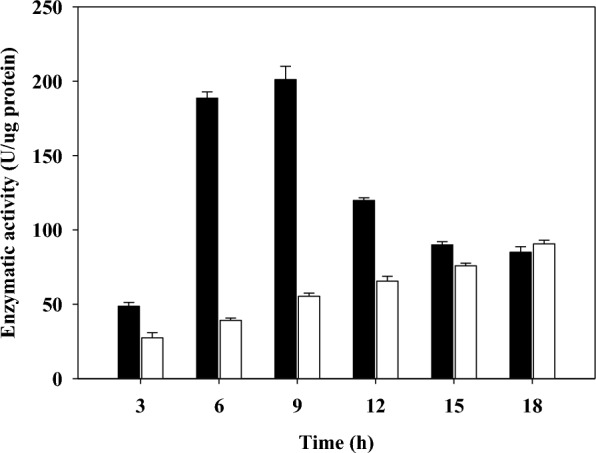
Redox enzymatic activity of acetoin reductase/2,3-butanediol dehydrogenase during batch fermentation of *Paenibacillus polymyxa* MDBDO. Black bars, acetoin reductase activity; open bars, 2,3-butanediol dehydrogenase activity

Interestingly, the amount of *R,R*-2,3-BDO gradually decreased in both strains after 12 h, at which time the amount of acetoin gradually increased. Also at 12 h, cell growth entered a stationary phase or death phase due to the depletion of the carbon source. We measured the enzymatic activity of AR/2,3-BDH of *P. polymyxa* MDBDO on production of acetoin and *R,R*-2,3-BDO during batch fermentation (Fig. 4). When the carbon source in the fermentation medium was sufficient (up to 12 h), the reducing activity of this enzyme increased gradually; however, upon depletion of the carbon source (after 12 h) the reducing activity declined and the oxidative activity gradually increased.

In general, AR/2,3-BDH has a higher substrate affinity for acetoin than *R,R*-2,3-BDO [39], but substrate affinity changes as the fermentation environment changes. After depletion of the carbon source in the fermentation broth, the cells require a new energy source. This energy can be obtained via oxidative phosphorylation [47], but this requires NADH [48]. Therefore, cells need NADH for survival after depletion of the carbon source from the fermentation broth. The reversible enzyme AR/2,3-BDH catalyzes *R,R*-2,3-BDO to R-2-acetoin, and converts the NAD^+^ produced during *R,R*-2,3-BDO production into NADH. Thus, depletion of the carbon source from the fermentation broth decreased the production of *R,R*-2,3-BDO and increased the production of acetoin. To our best knowledge, this study is the first report of a relationship between the carbon source and the reversible reaction of AR/2,3-BDH in the production of *R,R*-2,3-BDO.

The dissolved oxygen (DO) in the fermentation broth also decreases as cell growth increases, but it increases rapidly after depletion of the carbon source. During the fermentation of *P. polymyxa*, cells consume oxygen through catabolism of the carbon source to produce adenosine triphosphate (ATP). Therefore, the DO remains low when the cells are using a carbon source for growth. However, when cell growth slows or stops because of depletion of the carbon source, the DO increases. Aerobic conditions promote the production of *meso*-2,3-BDO [36]. For this reason, many previous researchers attempted to control the DO by altering the agitation speed and aeration rate during growth [29, 36–38]. Our results, similar to those of Hassler et al. [29], showed that *meso*-2,3-BDO was not produced due to low level of oxygen during fermentation, and the increase in DO after carbon source depletion also did not affect *meso*-2,3-BDO production. It is important to control the DO of the fermentation broth by adjusting agitation speed and aeration rate, but it is also important to supply an appropriate concentration of a carbon source so the cells consume available oxygen. Therefore, an appropriate carbon source is needed to maintain the reducing activity of AR/2,3-BDH and low oxygen concentration in the fermentation broth.

### Production of highly pure *R,R*-2,3-BDO using fed-batch fermentation with controlled carbon feeding

**Fig. 5:**
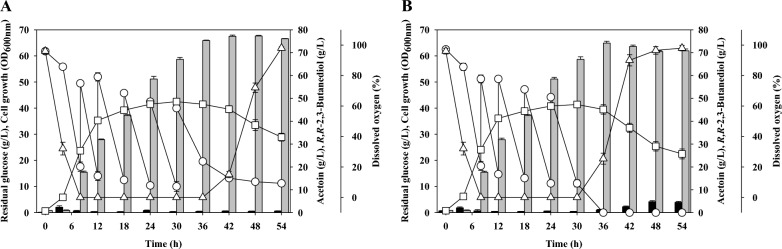
Fed-batch fermentation of *Paenibacillus polymyxa* MDBDO with a sufficient carbon supply (**A**) and an insufficient carbon supply (**B**). Open circles, residual glucose; open squares, cell growth; open triangles, dissolved oxygen; black bars, acetoin; gray bars, *R,R*-2,3-butanediol

Fed-batch fermentation can efficiently produce a high concentration of a desired product without substrate inhibition by intermittently or continuously supplying appropriate concentrations of substrate(s) [49]. This method prevents depletion of the carbon source and maintains the oxygen concentration at a low level due to cellular respiration during growth. We performed fed-batch fermentation to produce highly pure *R,R*-2,3-BDO using two carbon supply strategies: sufficient feeding (Fig. 5A) and limited feeding (Fig. 5B). During sufficient feeding, the carbon source was maintained above 10 g/L at the end of the fermentation, but during limited feeding the carbon source was completely depleted at 36 h. The maximal cell growth at 30 h was OD_600 nm_ 42.5 for sufficient feeding and OD_600 nm_ 41.4 for limited feeding. Both strategies led to similar cell growth because a carbon source was sufficiently supplied during the exponential growth phase. After 24 h, the carbon supply in the fermentation broth was sufficient, but cell growth did not increase further. Instead, cells remained in the stationary phase, and then gradually entered the death phase. During the growth of microorganisms, carbon is necessary for ATP production and nitrogen is necessary for diverse structural and functional molecules. Therefore, to increase cell concentration a carbon source and a nitrogen source are both essential [43, 50]. However, because these experiments did not supply a nitrogen source, the cells stopped grow despite the presence of a sufficient carbon source [51].

When using the sufficient feeding strategy, the maximal *R,R*-2,3-BDO production was 77.3 g/L at 48 h, and the amount of acetoin at that time was 0.6 g/L (Fig. 5A). *Meso*-2,3-BDO was undetectable, and the optical purity of *R,R*-2,3-BDO among C_4_ products (*R,R*-2,3-BDO, *meso*-2,3-BDO, and acetoin) was 99.2% (w/w). The DO in the fermentation broth declined sharply at the beginning of fermentation, and remained low during the exponential growth period, but it increased during stationary phase and death phase. Thus, as the cells gradually stopped growing, carbon consumption decreased and DO increased. An increased level of DO could have led to the production of *meso*-2,3-BDO, but *P. polymyxa* MDBDO did not produce this molecule. Through carbon supply control, *P. polymyxa* MDBDO produced *R,R*-2,3-BDO with the highest purity among non-GMO *Paenibacillus* sp. reported so far (Additional file 1: Table S1).

As a facultative anaerobe, *Paenibacillus* can grow in the presence or absence of oxygen. In the presence of oxygen, respiration produces ATP and this supports cell growth, but decreases the production of 2,3-BDO. On the other hand, in oxygen-limited conditions, there is increased production of 2,3-BDO, but respiration is inhibited and this leads to decreased cell growth. When using the sufficient feeding strategy, the DO increased after 36 h (Fig. 5A), and there was very little carbon uptake at that time and cells were therefore unable to produce *meso*-2,3-BDO. In addition, the low DAR activity in the mutant also inhibited *meso*-2,3-BDO production (Fig. 2). In a similar study, fed-batch fermentation of *P. polymyxa* DSM365 using 30 g/L yeast extract led to production of 64.0 g/L of *R,R*-2,3-BDO, 2.4 g/L of *meso*-2,3-BDO, and 9.5 g/L of acetoin at 48 h [29], and the purity of *R,R*-2,3-BDO among C_4_ products was only 84.3% (w/w).

The maximal *R,R*-2,3-BDO production using the limited feeding strategy was 74.2 g/L at 36 h, and the amount of acetoin at that time was 1.2 g/L (Fig. 5B). As with the sufficient feeding strategy, *meso*-2,3-BDO was undetectable. The DO change also had a similar trend in the limited and sufficient feeding strategies. However, during limited feeding, the cell concentration decreased more rapidly at the beginning, the production of acetoin increased, and the production of *R,R*-2,3-BDO decreased after 36 h. At 48 h the acetoin level was 4.8 g/L and the *R,R*-2,3-BDO level was 70.5 g/L. These results were similar to what occurs when a carbon source is depleted during batch fermentation, but the fraction of acetoin converted to *R,R*-2,3-BDO was lower than occurred during batch fermentation. During the limited feeding strategy, cells had reduced metabolic activities and entered the death phase at 36 h. This explains the lower acetoin conversion into *R,R*-2,3-BDO compared with batch fermentation. During the limited feeding fed-batch fermentation, the carbon source was depleted and the DO increased, but there was no *meso*-2,3-BDO production because there was no carbon available to produce its precursors.

### Production of *R,R*-2,3-BDO using CSL as alternative nitrogen source

A yeast extract can be used as a nitrogen source to produce *R,R*-2,3-BDO using *P. polymyxa* MDBDO, but this increases the production costs. Thus, we replaced the yeast extract with CSL, a low-cost industrial by-product that contains nitrogen. The yeast extract contained 10.3% (w/v) total nitrogen and our CSL contained 4.0% (w/v) total nitrogen [43]. Therefore, we used 75 g/L of CSL as a replacement for the 30 g/L of yeast extract, and then performed the sufficient feeding strategy described above for fed-batch fermentation experiment.

**Fig. 6:**
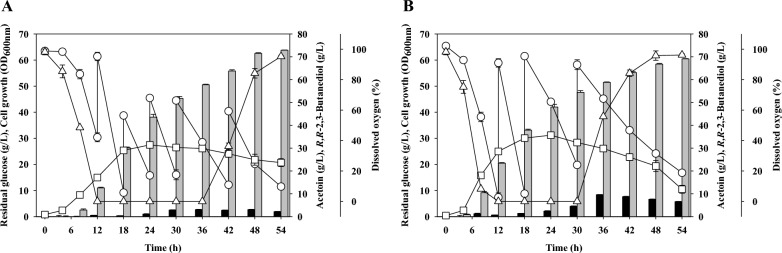
Fed-batch fermentation of *Paenibacillus polymyxa* MDBDO with a sufficient carbon supply using corn steep liquor as the sole nitrogen source (**A**) and using the 2,3-butanediol production medium with 60 mM lactic acid (**B**). Open circles, residual glucose; open squares, cell growth; open triangles, dissolved oxygen; black bars, acetoin; gray bars, *R,R*-2,3-butanediol

Use of fed-batch fermentation with sufficient feeding led to maximal cell growth of OD_600 nm_ 27.5 at 24 h, maximal *R,R*-2,3-BDO production of 72.9 g/L at 54 h an acetoin level of 2.2 g/L 54 h, and no detectable *meso*-2,3-BDO (Fig. 6). Unfortunately, the maximal cell growth and *R,R*-2,3-BDO production were less than achieved by fed-batch fermentation using yeast extract, and the amount of acetoin was greater. The CSL used in these experiments contained 7.2% lactic acid [43]. Thus, the fermentation broth that had the modified 2,3-BDO production medium (with CSL) had a lactic acid level of 60 mM. Nakashimada et al. examined the effects of various organic acids on the production of 2,3-BDO [37]. They found that when 100 mM lactic acid was added in batch fermentation, there was no significant increase in the production of 2,3-BDO compared to the control, but the level of acetoin decreased. Moreover, the addition of an organic acid decreased the growth of *P. polymyxa* in a concentration-dependent manner.

Thus, to determine the possible reason for the decreased cell growth and *R,R*-2,3-BDO production when CSL was the sole organic nitrogen source, we added 60 mM lactic acid to the 2,3-BDO production medium for fed-batch fermentation (Fig. 6B). The results showed that cell growth and *R,R*-2,3-BDO production decreased, similar to that when we used CSL as a nitrogen source. In particular, the initial lag phase of cell growth was longer than using the 2,3-BDO production medium, and the production of acetoin was greater than when CSL was used as alternative nitrogen source. These results show that lactic acid was likely responsible for the decreased *R,R*-2,3-BDO production when CSL was used as a nitrogen source. Nonetheless, our results confirmed that CSL has potential for use as an alternative nitrogen source for the production of a high-concentration of *R,R*-2,3-BDO using *P. polymyxa* MDBDO*.*

### Effect of *P. polymyxa* MDBDO fed-batch fermentation broth on plant growth

**Fig. 7:**
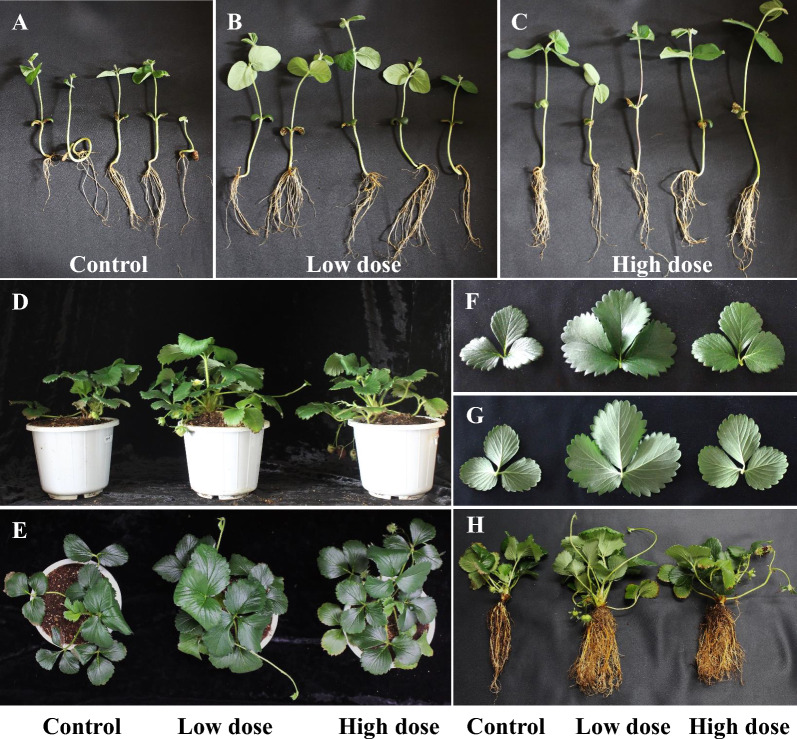
Effect of low-dose and high-dose fed-batch fermentation broth from *Paenibacillus polymyxa* MDBDO on growth of soybean seedlings (**A**–**C**) and strawberry seedlings (**D**–**H**). Control: distilled water

We next determined the effect of different doses of the *P. polymyxa* MDBDO fed-batch fermentation broth on the growth of soybean and strawberry seedlings. Both doses of this fermentation broth increased the mean shoot length and root length of soybean seedlings compared to the control treatment (distilled water) (Table 2, Fig. 7 A–C). Because of the different growth forms of soybean and strawberry, we measured strawberry’s leaf parameters (number of leaves, leaf length, and leaf width) and the fresh and dry weight of roots and shoots (Table 3, Fig. 7D–G). Similar to the soybean experiments, low-dose and high-dose fermentation broth significantly increased the number of leaves, leaf length, and leaf width, and also significantly increased the fresh and dry weights of shoots and roots (Table 4, Fig. 7H).

**Table 2:** Effect of low-dose and high-dose *Paenibacillus polymyxa* MDBDO fermentation broth on the growth of roots and shoots of soybean seedlings

Tissues	Treatment		
	Control	Low-dose	High-dose
Shoot (cm)	8.50 ± 0.93 b	14.80 ± 1.32 a	16.20 ± 2.09 a
Root (cm)	9.02 ± 0.80 b	12.00 ± 0.79 a	13.50 ± 0.68 a

Control: distilled water

Here and in Tables 3 and 4, data are given as means ± SEMs

In each row, values with different lowercase letters were significantly different in a pairwise comparison (*p* < 0.05)

**Table 3:** Effect of low-dose and high-dose *Paenibacillus polymyxa* MDBDO fermentation on the average number of leaves, leaf length, and leaf width of strawberry seedlings

Leaf characters	Treatment		
	Control	Low-dose	High-dose
Leaves (ea)	18.67 ± 0.67 b	24.00 ± 1.73 a	22.00 ± 1.00 ab
Length (mm)	38.33 ± 0.97 b	44.01 ± 1.02 a	40.04 ± 0.90 b
Width (mm)	30.26 ± 0.82 b	35.51 ± 0.95 a	32.43 ± 0.75 b

Control: distilled water

**Table 4:** Effect of low-dose and high-dose *P. polymyxa* MDBDO fermentation broth on the fresh weight and dry weight of strawberry seedlings

Treatment	Fresh weight (g)		Dry weight (g)	
	Shoot	Root	Shoot	Root
Control	5.77 ± 0.07 b	6.27 ± 0.27 b	1.20 ± 0.04 b	0.86 ± 0.08 b
Low-dose	10.35 ± 1.01 a	11.43 ± 0.56 a	3.78 ± 0.84 a	2.68 ± 0.19 a
High-dose	8.80 ± 0.61 a	9.38 ± 1.56 ab	2.65 ± 0.10 b	2.67 ± 0.59 a

Control: distilled water

Previous research also reported that the *P. polymyxa* fermentation broth increased plant growth (shoot length, root length, and biomass) in soybean, canola, kiwifruit, pepper, watermelon, rice, maize, potato, cucumber, beans, tomato, barley, *Arabidopsis*, and other ornamental plants [7, 51, 52]. It is also known that the 2,3-BDO secreted by bacteria stimulates root development, leading to increased uptake of nutrients and water and increased plant growth [4, 53]. 2,3-BDO is an essential organic component that functions in airborne chemical signaling, and it promotes growth in plants such as *Arabidopsis thaliana* [5]. A previous study reported that *Bacillus subtilis* produced organic volatile compounds that induced systemic resistance in Arabidopsis, and a *B. subtilis* mutant that had reduced 2,3-BDO production had a weaker effect [5]. These previous studies demonstrated the effect of microbial *R,R*-2,3-BDO in promoting plant growth. In agreement, the present study demonstrated that the *P. polymyxa* MDBDO fermentation broth, which contained highly pure *R,R*-2,3-BDO, promoted the growth of seedling shoots and roots, and increased plant biomass. Therefore, *P. polymyxa* MDBDO has potential application as a source for plant growth promoting agent.

## Conclusions

We demonstrated that *P. polymyxa* MDBDO, which we obtained by gamma-ray mutagenesis, had increased cell growth and AR/2,3-BDH activity compared to the parent strain. Growth of these cells in fed-batch fermentation with a controlled carbon concentration led to production of highly pure *R,R*-2,3-BDO, with very little acetoin production. To our best knowledge, we achieved a high concentration of *R,R*-2,3-BDO production with highest optical purity (> 99%) than previously reported for non-GMO *P. polymyxa* strains. In addition, the possibility of CSL as an alternative nitrogen source for the production of high-concentration *R,R*-2,3-BDO was confirmed. The *P. polymyxa* MDBDO fermentation broth also significantly promoted the growth of soybean and strawberry seedlings. This study suggests that *P. polymyxa* MDBDO has potential for use in various industrial applications.

## Methods

### Materials and growth media

Analytical standards of *R,R*-2,3-BDO, *meso*-2,3-BDO, and acetoin dimer were purchased from Sigma-Aldrich (St Louis MO, USA). Acetoin monomers were prepared by incubation of dimers at 95 °C for 1 h. CSL was purchased from Samyang Genex (Incheon, Korea). YPD (10 g/L yeast extract, 20 g/L peptone, 20 g/L glucose) medium was used for pre-activating and strain preservation. A previously described growth medium was modified and used as the 2,3-BDO production medium [29]. This medium contained 60 g/L glucose, 30 g/L yeast extract, 0.2 g/L MgSO_4_, 3 g/L (NH_4_)_2_SO_4_, 1.2 g/L KH_2_PO_4_, 0.2 g/L Na_2_HPO_4_, and 3 mL of trace element solution. The trace element solution contained 0.4 g/L FeSO_4_, 5.0 g/L MnCl_2_·4H_2_O, 0.1 g/L ZnSO_4_·7H_2_O, 0.8 g/L H_3_BO_3_, 0.04 g/L CuSO_4_·5H_2_O, 0.04 g/L NaMoO_4_·2H_2_O, 0.08 g/L Co(NO_3_)_2_·6H_2_O, 1.0 g/L CaCl_2_·2H_2_O, 0.01 g/L biotin, and few drops of HCl. All other chemicals were of analytical grade.

### Gamma-ray mutagenesis

The *P. polymyxa* DSM365 stock vial was inoculated in 10 mL of the 2,3-BDO production medium and activated on a shaker at 200 rpm at 37 °C for 36 h. The activated cells were then inoculated (10% v/v) in 40 mL of the 2,3-BDO production medium, then incubated on a shaker at 200 rpm at 37 °C for 12 h. After centrifugation at 4000 rpm at 4 °C for 10 min, the sample was washed with sterile phosphate-buffered saline (PBS, pH 6.0). For gamma-irradiation, the cells were resuspended in 10 mL of sterile PBS buffer. Gamma-irradiation was performed at the Korea Atomic Energy Research Institute (Jeonbuk, Korea), with gamma-ray doses of 1.0, 2.0, and 3.0 kGy. After irradiation, the cells were diluted, 100 µL was spread on a 2,3-BDO production plate, and the plate was incubated at 37 °C for 2 days. Colonies that were larger than the wild type strain were selected, and were subcultured three times on 2,3-BDO production plates to assure stability and consistency.

### Fermentation of *P. polymyxa* MDBDO

Evaluation of the mutant strains and preparation of seed cells for batch and fed-batch fermentation were performed in 1 L flasks containing 300 mL of the 2,3-BDO production medium. Flasks were incubated on a shaker at 200 rpm at 37 °C for 12 h, and cultures were subsequently inoculated into 5 L fermentors at a concentration of 10% (v/v). Batch fermentation was conducted in a 5 L stirred-vessel system (BIOCNS, Daejeon, Korea) that contained 3 L of the 2,3-BDO production medium. Unless otherwise stated, fermentation experiments were performed on a shaker at 500 rpm and 0.2 vvm at 37 °C, and the pH was maintained at 6.0 ± 0.1 using 28% (w/v) NH_4_OH and 2 M HCl.

Fed-batch fermentation was conducted in a 5 L stirred-vessel system that contained 3 L of the 2,3-BDO production medium or a modified 2,3-BDO production medium (75 g/L of CSL instead of 30 g/L yeast extract). The fed-batch fermentation used a solution containing 900 g/L glucose as the feed, and feeding was intermittent to maintain the glucose concentration in the fermentation broth. Cells were sampled at different times for enzyme activity assays, and the metabolites in fermentation supernatants were analyzed. Cell growth was monitored by measurement of OD_600 nm_ using a UV–Vis spectrophotometer (Ultrospec 3100 Pro; Amersham Biosciences, NJ, USA). All results are averages from two independent experiments.

### High-performance liquid chromatography

Residual substrates and fermentation metabolites were analyzed using a high-performance liquid chromatography system (Agilent 1260) that was equipped with a refractive index detector and an Aminex HPX-87H column (300 × 78 mm; Bio-Rad, CA, USA). The mobile phase was 2.5 mM H_2_SO_4_, the flow rate was 0.6 mL/min, the column temperature was 65 °C, and the cell temperature was 45 °C [54]. Analysis samples were analyzed after centrifugation at 13,000 rpm for 10 min and then filtered with 0.22um.

### Enzyme activity

*P. polymyxa* DSM365 and *P. polymyxa* MDBDO cells were harvested by centrifugation and washed two times with 33 mM sodium phosphate (pH 6.0). Cells were disrupted using an ultrasonic system for 1 min, using cycles of crushing for 2 s and resting 6 s (Power: 30%, 210 W, 19,736 Hz) in the same buffer. A crude extract was obtained by centrifugation 13,000 rpm for 10 min at 4 °C. Protein concentration of crude extract was determined using the Bradford assay, with bovine serum albumin as the standard. The reducing activity of AR was determined by measuring OD_340 nm_ at 37 °C (corresponding to a decrease of NADH). The activity of the crude extract was measured with 50 mM acetoin and 4 mM NADH in 33 mM sodium phosphate (pH 6.0). The oxidation activity of *R,R*-2,3-BDH was determined by measuring OD_340 nm_ at 37 °C (corresponding to an increase of NADH). The activity of the crude extract was measured using 50 mM *R,R*-2,3-BDO and 4 mM NAD^+^ in 33 mM sodium phosphate (pH 6.0).

The activity of DAR was determined by measuring OD_340 nm_ at 37 °C (corresponding to a decrease of NADPH). The activity of the crude extract was measured using 50 mM butanedione and 4 mM NADPH in 33 mM sodium phosphate (pH 6.0).

One unit of AR/2,3-BDH activity and 1 unit of DAR activity corresponded to the formation of 1 mol of each product (NAD^+^, NADH, or NADP^+^) per min, and enzymatic activity was expressed U/µg protein. All results are averages from two independent experiments.

### Effect of *P. polymyxa* MDBDO fermentation broth on plant growth

The effect of the *P. polymyxa* MDBDO fed-batch fermentation broth (48 h), which was established by using sufficient carbon source, on the growth of soybean (cv. Daewon) and strawberry (cv. Sulhyang) seedlings was determined. Two different doses (low dose: 50-fold dilution, high dose: fivefold dilution) of the fermentation broth were added to the potted plants at a rate of 1 L/m^2^ (soybean: 10 mL/pot, strawberry: 30 mL/pot), and control plants received distilled water. For soybean, two doses were applied to the potting soil at one week intervals at 7 days after seed sowing. After 2 weeks of seedling growth, plant height (shoot) and root length were measured using a vernier caliper. There were five seeds per pot, and three pots per treatment. For strawberry, seedlings were collected from a nursery bed and transferred to potting soil. The two doses of the fermentation broth were applied at one week intervals. There was one seedling per pot and three pots per treatment. At two weeks after the final treatment, the number of leaves, leaf surface area, and shoot and root biomass (fresh and dry weight) were measured. Soybean and strawberry seedlings were cultivated at a temperature of 25 °C and under a 16/8 light–dark cycle, and were watered whenever necessary.

## Supplementary Information

**Additional file 1: Table S1.** Comparison of production and optical purity of *R,R*-2,3-butanediol by various *Paenibacillus *strains.

## Data Availability

All data generated or analyzed during this study are included in this published article [and its Addiitonal files].
