# Supplementary material for: Production of highly pure R,R-2,3-butanediol for biological plant growth promoting agent using carbon feeding control of Paenibacillus polymyxa MDBDO
Source: Microb Cell Fact. 2023 Jul 5;22:121. doi: 10.1186/s12934-023-02133-y (PMC10320955; doi:10.1186/s12934-023-02133-y)
Supplement: Supplementary file 1 — Additional file 1: Table S1. Comparison of production and optical purity of R,R-2,3-butanediol by various Paenibacillus strains. [file 12934_2023_2133_MOESM1_ESM.docx]

**Table S1** Comparison of production and optical purity of *R,R*-2,3-butanediol by various *Paenibacillus* strains.

| **Strain** | **Genetic**  **modification** | ***R,R*-2,3-BDO**  **production (g/L)** | ***R,R*-2,3-BDO**  **productivity (g/L)** | ***R,R*-2,3-BDO purity**  **(% of C_4_ products ^a^)** | **Reference** |
| --- | --- | --- | --- | --- | --- |
| *P. polymyxa* ZJ-9 | Non-GOM | 36.9 | 0.88 | 98.1 ^b^ | [8] |
| *P. polymyxa* ZJ-9 | GOM | 25.9 | 0.43 | 99.9 | [19] |
| *P. polymyxa* DSM365 | Non-GOM | 111.0 | 2.06 | 98.2 | [24] |
| *P. polymyxa* DSM365 | Non-GOM | 72.0 | 1.33 | 79.0 | [24] |
| *P. polymyxa* ATCC12321 | Non-GOM | 9.6 | 0.20 | 97.1 | [32] |
| *P. polymyxa* MDBDO | Non-GOM | 77.3 | 1.61 | 99.2 | This study |

^a^ C_4_ products: *R,R*-2,3-BDO, *meso*-2,3-BDO, and acetoin.

^b^ Acetoin data not available.
